# Meropenem treatment failure in severe melioidosis despite in vitro susceptibility: A case report

**DOI:** 10.1016/j.idcr.2026.e02648

**Published:** 2026-06-19

**Authors:** Bui Hai Hoang, Dinh Thang Ba, Tuan Viet Luu, Dinh Hung Vu, Anh Dung Nguyen, Thi Huong Thao Bui, Thi Dieu Ngan Ta, Aurelien Dinh

**Affiliations:** a Hanoi Medical University, Hanoi, Viet Nam; b Emergency and Critical Care Department, Hanoi Medical University Hospital, Hanoi 100000, Viet Nam; c Department of Tropical Diseases and Harm Reduction, Hanoi Medical University Hospital, No.1, Ha Noi 100000, Viet Nam; d Pharmacology Department, Hanoi Medical University, Viet Nam; e Infectious disease department, R. Poincaré University Hospital, APHP, Garches, France

**Keywords:** Melioidosis, *Burkholderia pseudomallei*, Meropenem treatment failure, Ceftazidime, Relapse, Case report

## Abstract

*Burkholderia pseudomallei* causes melioidosis, a potentially life-threatening infection that can rapidly progress to septic shock and multi-organ failure if not promptly recognized and treated. Current international guidelines recommend meropenem as first-line therapy for severe melioidosis, whereas prolonged oral eradication therapy is essential to prevent relapse. We report a 39-year-old man with diabetes mellitus and chronic hepatitis B who initially improved on intravenous ceftazidime for disseminated melioidosis, but relapsed after an inadequate eradication phase consisting of only one week of oral amoxicillin-clavulanate. During the second admission, he presented with septic shock and multiple deep abscesses. He initially stabilized on combination therapy including meropenem and ceftazidime, but deteriorated after de-escalation to meropenem monotherapy despite repeated in vitro susceptibility. Clinical improvement occurred after reintroduction and continuation of high-dose ceftazidime together with drainage of accessible abscesses. This case highlights that apparent clinical failure on meropenem monotherapy may be confounded by inadequate eradication therapy, incomplete source control, borderline baseline susceptibility, and possible pharmacokinetic factors. It also underscores the need to interpret in vitro susceptibility cautiously in severe relapsed melioidosis.

## Highlights

• Severe melioidosis showed clinical failure under meropenem despite susceptibility.
• Rapid clinical and microbiological response occurred after switching to ceftazidime.
• In vitro susceptibility did not predict therapeutic response to meropenem.
• Combination therapy was required to achieve sustained clinical stabilization.

## Introduction

Melioidosis is a major cause of community-acquired pneumonia and bacteraemia in South-East Asia and northern Australia, but remains rarely diagnosed in travellers returning from endemic regions [1]. The disease is caused by *Burkholderia pseudomallei*, a Gram-negative environmental bacterium found in soil and surface water in tropical and subtropical areas. Clinical presentations are highly variable and range from localized infection to severe disseminated disease, frequently complicated by abscess formation and multi-organ involvement. Host-related risk factors associated with severe disease include diabetes mellitus, chronic lung disease, and chronic kidney injury [2].

*B. pseudomallei* exhibits intrinsic resistance to multiple antibiotic classes, posing significant therapeutic challenges. Although acquired resistance may occasionally emerge during antimicrobial therapy, the absence of human-to-human transmission limits the spread of resistant strains. Current management of melioidosis relies on a two-phase antibiotic strategy, consisting of an intensive intravenous treatment during the acute phase, followed by prolonged oral eradication therapy to prevent relapse [3], [4]. High-dose ceftazidime has demonstrated efficacy as initial therapy in randomized clinical trials and remains an option for non-critically ill patients [5]. However, observational data suggest superior outcomes with carbapenems in severe disease, and meropenem is therefore widely regarded as the treatment of choice for critically ill patients with melioidosis [6], [7], [8]. Notably, prospective comparative trials evaluating carbapenems versus third-generation cephalosporins remain scarce [9].

Despite the generally preserved susceptibility of *B. pseudomallei* to carbapenems, the emergence of carbapenem resistance during treatment has been reported [1].

In this report, we describe a patient with disseminated melioidosis who initially improved with ceftazidime, later relapsed after inadequate oral eradication therapy, and then experienced clinical deterioration on meropenem monotherapy despite reported in vitro susceptibility. Rather than suggesting intrinsic meropenem resistance, this case highlights the multifactorial nature of treatment failure in severe relapsed melioidosis.

## Clinical case

A 39-year-old man with a medical history of chronic hepatitis B virus infection (on tenofovir therapy) and diabetes mellitus (insulin-treated for the past three years) was admitted to the hospital on September 5, 2025, for high-grade fever (up to 40 °C) and back pain. His symptoms, including lumbar and left paraspinal pain, had been evolving for eight days.

On physical examination, the patient was conscious, normotensive (115/75 mmHg), tachycardic (106 beats/min), and febrile (40 °C), with tenderness over the left flank. Initial laboratory investigations revealed a white blood cell count of 6.43 G/L, a C-reactive protein (CRP) level of 18.8 mg/dL, and poor glycaemic control (HbA1c 10.3%) (Table 1). Blood cultures were obtained at admission.

**Table 1:** Laboratory findings at ICU admission.

Parameter	Result	Reference range
White blood cells	18.46 G/L	4.0–10.0 G/L
Neutrophils	77.6%	40–75%
Platelets	132 G/L	150–400 G/L
Procalcitonin	> 100 ng/mL	< 0.05 ng/mL
Troponin T	36.3 ng/L	< 14 ng/L
Pro-BNP	24,626 pg/mL	< 125 pg/mL
Urea	15.7 mmol/L	2.5–7.5 mmol/L
Creatinine	247 µmol/L	62–106 µmol/L
AST	564 U/L	< 40 U/L
ALT	250 U/L	< 41 U/L
Blood lactate	5.5 mmol/L	0.5–2.0 mmol/L
HbA1c	10.3%	< 5.7%

**Fig. 1:**
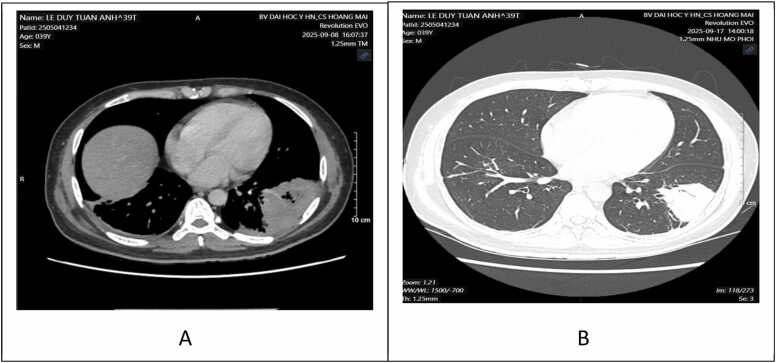
A) Chest CT scaner at the admission showed areas of consolidation at both lung with a large lesion containing a cavitary necrotic component on the left. B. Imaging on 11 days of treatment revealed a 44 × 41 mm mass in the left lower lobe, containing several fluid-filled bronchial branches, with heterogeneous enhancement after contrast administration (smaller lesion compared with image A).

During the first three days after admission, the patient received symptomatic treatment only, including paracetamol and non-steroidal anti-inflammatory drugs, because the initial presentation was not yet considered strongly suggestive of invasive bacterial infection. His clinical condition then worsened, with increasing asthenia and confusion. Chest computed tomography showed bilateral basal consolidative lesions, including a larger left-sided lesion with cavitary necrosis (Fig. 1**A**). Empirical intravenous ceftazidime was started.

**Fig. 2:**
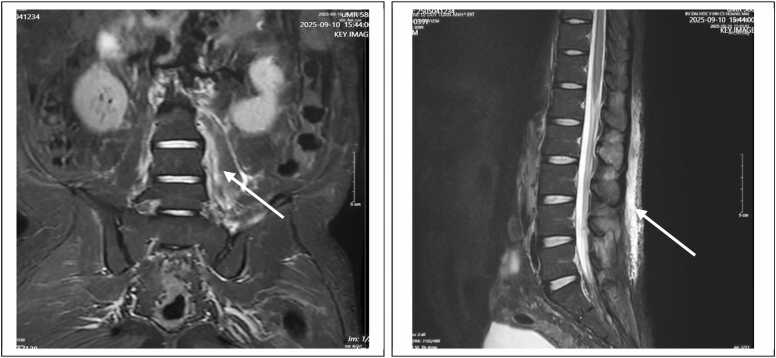
Contrast- enhanced MRI of the lumbar spine showing a diffuse edema with increased muscle volume and signal intensity was observed in the bilateral paraspinal muscles, iliopsoas muscles, and the lumbar paraspinal soft tissues (white arrow).

One day later, blood cultures grew *Burkholderia pseudomallei*. Positive blood culture bottles were processed using the Bact/Alert® 3D automated blood culture system (bioMérieux). Subcultures were performed on standard media and incubated at 37 °C for 24–48 h. Isolates were identified as B. pseudomallei by MALDI-TOF MS (Vitek MS, bioMérieux). Ceftazidime was continued at a dose of 8 g/day (2 g intravenously every 6 h), resulting in gradual clinical improvement over five days, with fewer febrile episodes (38–38.5 °C) and a decline in CRP levels. Ongoing lumbar pain prompted magnetic resonance imaging (MRI) of the lumbar spine, which demonstrated diffuse muscle oedema with increased muscle bulk and signal intensity involving the paraspinal muscles, iliopsoas muscles, and bilateral lumbar soft tissues, consistent with myositis (Fig. 2).

By Day 14, the patient became afebrile and follow-up blood cultures were negative. A repeat chest CT on Day 15 showed regression of pulmonary inflammatory lesions (Fig. 1B). Intravenous ceftazidime was given for a total of 29 days. At discharge after the first hospitalization, oral amoxicillin-clavulanate was prescribed for only one week. This course was substantially shorter than recommended eradication therapy for melioidosis.

negative, and a repeat chest CT scan performed 11 days after treatment initiation showed regression of pulmonary inflammation(Fig. 1B). Intravenous ceftazidime was administered for a total duration of 29 days. Upon completion of intravenous therapy, oral amoxicillin–clavulanate was initiated at a daily dose of 2000 mg for one week.

At the end of this treatment course, the patient developed chest pain and progressively worsening dyspnoea and was readmitted to hospital. Imaging revealed a persistent lung abscess, and treatment with cefoperazone–sulbactam combined with ofloxacin was prescribed for 10 days without durable improvement. Fever and asthenia recurred.

On Day 1 of the second hospitalization, the patient was admitted to the intensive care unit with septic shock, somnolence, delirium, tachypnoea, and hypoxaemia (SpO₂ 90%). Initial management included aggressive fluid resuscitation, oxygen supplementation, intravenous ceftazidime, and linezolid; linezolid was added empirically because of concern for a possible concomitant nosocomial Gram-positive infection. Owing to persistent hypotension (62/40 mmHg), norepinephrine infusion and mechanical ventilation were required. Meropenem was started at 2 g intravenously every 8 h (total daily dose 6 g/day) using standard intermittent infusion rather than extended infusion. Combination therapy with meropenem, ceftazidime, and linezolid was administered, and a dorsal soft tissue abscess was surgically drained.

**Fig. 3:**
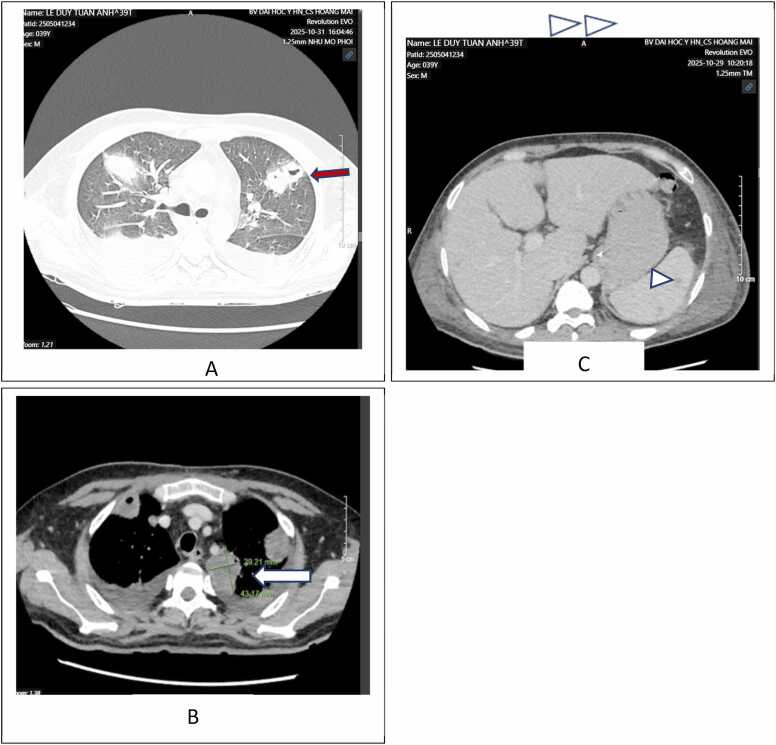
A) Contrast- enchanced CT images illustrating multiple lessions in the bilateral lung, with a centrall non enhancing fluid- density necrotic component (red arrow). B multiple loculated fluid collections within the bilateral paraspinal muscles at the L1–L5 levels, with partial communication with a collection in the left iliopsoas muscle (white arrow).C) Multiple hypodense nodules were scattered throughout the splenic parenchyma, showing poor enhancement after contrast administration white arrowhead).

Chest and abdominal CT scans revealed lung abscesses, pleural effusion, multiple abscesses involving the bilateral paraspinal soft tissues and left iliopsoas muscle, as well as new lesions in the hepatic and splenic parenchyma (Fig. 3).

Drainage fluid, sputum, and blood cultures remained positive for *B. pseudomallei*. Antimicrobial susceptibility testing was performed using the Vitek 2 Compact system (bioMérieux), and interpretation was made according to EUCAST breakpoints. However, no secondary confirmation by broth microdilution or Etest was available. A comparison of the susceptibility results from the two admissions is shown in Table 2.

**Table 2:** Antimicrobial susceptibility results of *B. pseudomallei* isolates.

Antibiotic	Isolate 1 MIC (µg/mL)	Isolate 1 interpretation	Isolate 2 MIC (µg/mL)	Isolate 2 interpretation
Ceftazidime	4	Susceptible	4	Susceptible
Imipenem	0.75	Susceptible	0.75	Susceptible
Meropenem	2	Susceptible	0.5	Susceptible
Trimethoprim-sulfamethoxazole	20	Susceptible	20	Susceptible

### MIC: minimum inhibitory concentration

*All isolates in this case were reported as susceptible to the agents listed.*

After seven days of combination antimicrobial therapy, the patient improved markedly: he became afebrile, vasopressor support was discontinued, he was successfully extubated, and follow-up blood cultures became negative. Because of apparent stabilization and in vitro susceptibility, ceftazidime was discontinued and meropenem monotherapy was continued (Table 3).

**Table 3:** Relative clinical timeline.

Three days after de-escalation to meropenem monotherapy, the patient relapsed clinically with recurrent fever (39 °C), agitation, and delirium. White blood cell count increased to 22.7 G/L, and repeat blood cultures again yielded *B. pseudomallei,* with a meropenem MIC of 0.5 µg/mL. Repeat imaging showed persistent pulmonary and iliopsoas abscesses without vertebral osteomyelitis. Given the suspected lack of clinical response to meropenem monotherapy, ceftazidime was reintroduced and combinated with doxycylin (100 mg twice a day)

Following an additional seven days of therapy, the patient stabilized, became afebrile, regained full consciousness, and required only minimal supplemental oxygen. Ceftazidime was subsequently continued for a further two weeks, leading to definitive clinical improvement and final discharge after a total treatment duration of 25 days.

After an additional seven days of combination therapy, the patient stabilized again, became afebrile, recovered full consciousness, and required only minimal supplemental oxygen. Ceftazidime was then continued for a further two weeks, leading to sustained improvement and final discharge. At final discharge, the patient was prescribed oral trimethoprim-sulfamethoxazole for a planned 3-month eradication phase with outpatient infectious diseases follow-up.

## Discussion

This case describes a patient with disseminated melioidosis who improved during the first admission on intravenous ceftazidime, relapsed after an inadequate oral eradication phase, and later deteriorated during meropenem monotherapy despite reported in vitro susceptibility. The central message of this case is not that meropenem intrinsically “failed,” but that apparent clinical failure on meropenem occurred within a highly confounded context that included inadequate eradication therapy, incomplete source control, severe disease burden, and possible pharmacokinetic limitations.

*Burkholderia pseudomallei* is highly prevalent in Southeast Asia and northern Australia, with Vietnam reporting a rising number of cases in recent years [7]. Our patient, who resided in Thanh Hoa Province—an area recognized as endemic—and presented with well-established risk factors for severe melioidosis, including poorly controlled diabetes mellitus and chronic hepatitis B infection, was appropriately diagnosed with melioidosis based on positive blood cultures for *B. pseudomallei*. This diagnosis was fully consistent with his clinical presentation, characterized by prolonged fever, back pain, and disseminated abscess formation involving the lungs and soft tissues.

Initial treatment with ceftazidime during the first hospitalization resulted in marked clinical and biological improvement, including resolution of fever, a significant decline in CRP levels, and clearance of bacteraemia. However, the absence of subsequent oral eradication therapy likely contributed to early relapse, which manifested as severe septic shock with multi-organ failure and the development of new abscesses involving the paraspinal soft tissues and hepatic parenchyma. During this second, life-threatening presentation, ceftazidime was promptly reintroduced, and meropenem was added in accordance with current recommendations for the management of severe septic melioidosis [2].

To date, no intrinsic resistance of *B. pseudomallei* to carbapenems has been documented, although resistance may occasionally be selected during ongoing treatment [9], [10]. In vitro susceptibility data from Thailand consistently demonstrate preserved activity of meropenem against *B. pseudomallei*, with approximately 98% of isolates remaining susceptible, alongside comparably high susceptibility rates to ceftazidime [11]. Despite this, data correlating microbiological susceptibility with clinical response in endemic regions remain limited. At the global level, meropenem resistance is exceedingly rare and is almost exclusively reported in patients with prior exposure to carbapenems [12], [13]. In the absence of any history of carbapenem therapy, the lack of clinical response observed in our patient highlights an exceptional presentation of melioidosis and underscores the possibility of primary treatment failure with meropenem, even in settings where resistance is considered highly uncommon.

The combined therapy was initially successful: the patient became afebrile, came off oxygen and vasopressors, and his inflammatory markers improved, with a subsequent negative blood culture. Crucially, when ceftazidime was discontinued and meropenem monotherapy was continued, the patient's clinical status deteriorated, and his blood culture reverted to positive for *Burkholderia pseudomallei*. This observation suggests the potential development of drug resistance or reduced susceptibility to meropenem during treatment. Hypothesized mechanisms for carbapenem resistance in *B. pseudomallei* include changes at the antibiotic target site and, notably, the activity of drug efflux pumps [2], [8]. Other authors have documented similar non-responses linked to gene mutations affecting efflux pump regulators [9]. However, in our case, the meropenem MIC was 2 μg/mL upon first admission and 0.5 μg/mL at the second admission, making common resistance mechanisms like *penA* gene mutation (which typically increases the MIC) less likely [9].

Previous studies have demonstrated the superiority of carbapenems over ceftazidime in the management of severe melioidosis [14], [15]. However, the present case highlights that, in situations of apparent clinical non-response to carbapenems, a therapeutic strategy based on high-dose ceftazidime should be actively considered. Notably, in vitro susceptibility testing did not demonstrate resistance to meropenem. Despite this, the patient’s clinical condition progressively deteriorated during seven days of meropenem therapy and showed a rapid and marked improvement following the switch to high-dose ceftazidime. The underlying mechanisms accounting for the discordance between in vitro susceptibility results and clinical treatment failure remain poorly understood. Previous work has described the emergence of growth-defective *B. pseudomallei* associated with ceftazidime treatment failure, potentially leading to false-negative susceptibility testing for resistant strains [16]. To date, a comparable phenomenon has not been reported for isolates associated with meropenem treatment failure.

The most important contributor to relapse was likely the inadequate eradication phase after the first hospitalization. Standard management of melioidosis requires an intensive intravenous phase followed by prolonged oral eradication therapy, usually for 3–6 months, most commonly with trimethoprim-sulfamethoxazole. In the present case, the patient received only one week of oral amoxicillin-clavulanate after 29 days of intravenous ceftazidime. This represents a major deviation from guideline-based care and likely played a central role in the subsequent relapse with septic shock and disseminated abscesses.

A second major confounding factor is source control. The patient had multiple deep-seated collections involving the lungs, paraspinal tissues, iliopsoas muscle, liver, and spleen. Although one dorsal abscess was drained, complete drainage of all collections was not achieved. In such a high-inoculum setting, persistent infection may occur despite administration of microbiologically active antibiotics. Accordingly, incomplete source control must be regarded as a plausible contributor to the deterioration observed during meropenem monotherapy.

Pharmacokinetic/pharmacodynamic considerations are also important. Meropenem was administered at 2 g every 8 h by standard intermittent infusion. Although this is a high dose, extended infusion was not used, and drug concentrations were not measured. In young critically ill patients with sepsis, augmented renal clearance may reduce beta-lactam exposure and prevent optimal time above the MIC. This is particularly relevant because the initial meropenem MIC was 2 µg/mL, which lies at the upper limit of the EUCAST susceptible breakpoint. Therefore, suboptimal tissue or serum exposure may have contributed to poor clinical response despite nominal in vitro susceptibility.

Active antibiotic efflux mediated by resistance–nodulation–cell division (RND) efflux pumps represents a major mechanism of carbapenem resistance in *B. pseudomallei* [9], [17]. Such overexpression typically emerges under antibiotic selective pressure and is rarely present in initial isolates. Although this mechanism may account for the observed clinical treatment failure, efflux pump expression was not assessed in the present case. Additionally, mutations in regulatory regions upstream of the *penA* gene can lead to overexpression of class A β-lactamase, conferring resistance to ceftazidime and reduced susceptibility to meropenem [18]. However, the favourable clinical response to high-dose ceftazidime makes a *penA*-mediated mechanism unlikely. While conclusions on antibiotic management are limited by the single-case design, this observation highlights the need for increased awareness and further investigation of carbapenem-unresponsive melioidosis.

The MIC trajectory in this case does not support emergent microbiological resistance. In fact, the meropenem MIC decreased from 2 µg/mL in the first isolate to 0.5 µg/mL in the second isolate. This finding is biologically inconsistent with acquisition of resistance during treatment and argues against the earlier interpretation that treatment failure reflected progressive meropenem resistance. Instead, the borderline baseline MIC, the limitations of the AST method, and clinical confounders together provide a more plausible explanation.

The AST methodology is another important limitation. Susceptibility testing was performed using Vitek 2 Compact, which is not the reference method for B. pseudomallei and may be unreliable for carbapenem testing. The isolates were not retested in parallel by broth microdilution or Etest, and no molecular analysis such as whole-genome sequencing or evaluation of efflux pump regulation was performed. These limitations prevent any definitive mechanistic conclusion regarding reduced carbapenem activity.

The absence of trimethoprim-sulfamethoxazole during the acute severe relapse also deserves comment. In many centers, TMP-SMX is combined with intravenous therapy in severe disseminated melioidosis. In this case, TMP-SMX was not initiated during ICU management because of acute kidney injury and concerns regarding additional nephrotoxicity. Although clinically understandable, this decision may also have influenced treatment response.

A similar case of melioidosis clinically unresponsive to meropenem has recently been reported by Grewe et al. [5]. Our case differs in several respects: the patient had no prior carbapenem exposure before the second admission, the reported meropenem MIC decreased rather than increased over time, and relapse occurred in the context of markedly inadequate eradication therapy and incomplete source control. These distinctions support the relevance of the present case while also tempering claims of novelty.

Overall, this case should be interpreted as a cautionary example of how severe relapsed melioidosis can mimic antimicrobial failure even when in vitro susceptibility is preserved. The case reinforces several practical lessons: the eradication phase is essential; source control must be aggressively pursued whenever feasible; PK/PD optimization should be considered in critically ill patients; and AST results should be interpreted cautiously when obtained with non-reference methods.

## Conclusion

This report describes clinical deterioration on meropenem monotherapy despite in vitro susceptibility in a patient with severe relapsed melioidosis. However, the observed non-response was likely multifactorial and strongly confounded by inadequate eradication therapy after the first admission, incomplete source control, severe disseminated disease, and possible suboptimal meropenem exposure. The main clinical lesson is the importance of adequate eradication therapy and careful interpretation of susceptibility data rather than a definitive claim of intrinsic meropenem failure.

## CRediT authorship contribution statement

**Thang Dinh:** Writing – review & editing, Validation, Methodology, Investigation. **Tuan Viet Luu:** Methodology, Investigation, Formal analysis. **Dinh Hung Vu:** Validation, Methodology, Investigation, Formal analysis. **Anh Dung Nguyen:** Writing – review & editing, Supervision, Investigation. **Bui Hai Hoang:** Writing – review & editing, Writing – original draft, Validation, Supervision, Methodology, Investigation, Formal analysis, Data curation, Conceptualization. **Dinh Aurélien:** Validation, Supervision, Formal analysis. **Thi Huong Thao Bui:** Writing – review & editing, Validation, Formal analysis, Conceptualization. **Thi Dieu Ngan Ta:** Writing – review & editing, Validation, Conceptualization.

## Authorship and contribution

All listed authors meet the authorship criteria as defined by the International Committee of Medical Journal Editors (ICMJE). Each author has made a substantial contribution to the conception, design, data acquisition, analysis, or interpretation of the work, has participated in drafting or critically revising the manuscript, and has approved the final version for submission.

## Patient consent

Written informed consent was obtained from the patient for publication of this case report and the accompanying images. A copy of the written consent is available for review by the Editor-in-Chief of this journal on request.

## Ethics statement

This case report was conducted in accordance with the ethical standards of Hanoi Medical University Hospital and the principles of the Declaration of Helsinki. Formal institutional review board approval was not required for a single-patient case report at our institution. Patient consent was obtained as stated above.

## Ethical considerations

The study was conducted in accordance with ethical standards. Informed consent for publication was obtained from the patient. Patient anonymity has been preserved, and no identifying information is disclosed in the manuscript.

## Funding

No external funding was received for this work.

## Declaration of Competing Interest

The authors declare that they have no known competing financial interests or personal relationships that could have appeared to influence the work reported in this paper.
